# Remodeling Brain Activity by Repetitive Cervicothoracic Transspinal Stimulation after Human Spinal Cord Injury

**DOI:** 10.3389/fneur.2017.00050

**Published:** 2017-02-20

**Authors:** Lynda M. Murray, Maria Knikou

**Affiliations:** ^1^Motor Control and NeuroRecovery Laboratory, Department of Physical Therapy, College of Staten Island, New York, NY, USA; ^2^Departments of Neuroscience and Biology, Graduate Center, City University of New York, New York, NY, USA

**Keywords:** cortical plasticity, corticospinal plasticity, primary motor cortex, repetitive transspinal stimulation, spinal cord injury

## Abstract

Interventions that can produce targeted brain plasticity after human spinal cord injury (SCI) are needed for restoration of impaired movement in these patients. In this study, we tested the effects of repetitive cervicothoracic transspinal stimulation in one person with cervical motor incomplete SCI on cortical and corticospinal excitability, which were assessed *via* transcranial magnetic stimulation with paired and single pulses, respectively. We found that repetitive cervicothoracic transspinal stimulation potentiated intracortical facilitation in flexor and extensor wrist muscles, recovered intracortical inhibition in the more impaired wrist flexor muscle, increased corticospinal excitability bilaterally, and improved voluntary muscle strength. These effects may have been mediated by improvements in cortical integration of ascending sensory inputs and strengthening of corticospinal connections. Our novel therapeutic intervention opens new avenues for targeted brain neuromodulation protocols in individuals with cervical motor incomplete SCI.

## Introduction

In the last two decades, neuromodulation protocols that utilize electromagnetic stimulation have been developed with the aim to produce functional neuroplasticity and recovery of motor function after upper motoneuron lesions in humans. One representative neuromodulation protocol is that of electromagnetic stimulation delivered to the primary motor cortex (M1). Specifically, repetitive transcranial magnetic stimulation (rTMS) delivered at stimulation frequencies ranging from 3 to 5 Hz increased the amplitude of the motor-evoked potentials (MEPs) recorded from distal and proximal arm muscles (1). By contrast, rTMS delivered at 0.9 Hz for 15 min decreased the MEPs recorded from arm muscles while at rest (2).

In this context, repetitive non-invasive transspinal stimulation may constitute a novel therapeutic strategy to strengthen corticospinal connections after spinal cord injury (SCI) in humans. Primate and animal models of SCI have showed marked spontaneous plasticity of corticospinal projections driven partly from sprouting of spinal cord midline crossing axons and *via* reorganization of propriospinal connections (3, 4). The longer latencies and higher thresholds of MEPs in people with motor incomplete SCI (5), related partly to degeneration and atrophy of the axons distal from the injury site (6), support the need for developing neuromodulation protocols that can strengthen corticospinal connections in these individuals. Moreover, the well-documented bilateral projection of corticospinal axons in the gray matter of the primate spinal cord (7), regeneration of injured spinal cord neurons in response to electrical fields (8), and potentiation of intracortical facilitation (ICF) following spinal cord stimulation for pain (9) further support the use of repetitive transspinal stimulation as a strategy to strengthen corticospinal connections.

Electrical stimulation delivered transcutaneously to the spinal cord at cervicothoracic or thoracolumbar regions generates transspinal-evoked potentials in proximal and distal arm and leg muscles simultaneously with distinct neurophysiological characteristics. This form of stimulation produces a marked modulation of neuronal excitability at cortical, corticospinal, and spinal levels when delivered alone or when paired with TMS over the M1 (10–15). More importantly, the summation of transspinal-evoked potentials with the homonymous MEPs suggests that transspinal stimulation can directly affect the activity of corticospinal axons (13). Lastly, non-invasive transspinal stimulation entrains the motor output of previously silent muscles during robotic-assisted stepping in people with SCI (16). However, the effects of repetitive transspinal stimulation on neuronal excitability in people with SCI have not been investigated. In this study, we assessed the effects of repetitive transspinal stimulation over the cervicothoracic region on cortical and corticospinal excitability in a person with chronic cervical motor incomplete SCI. We hypothesized that transspinal stimulation strengthens corticospinal connections, reorganizes activity of cortical neural circuits, and improves arm motor function.

## Materials and Methods

### Participant

One person (27 years, male) with an injury at cervical 6–7 due to a motor vehicle crash [American Spinal Injury Association Impairment Scale (AIS) C for upper extremities and AIS B for lower extremities], 9-year post-injury participated in the study. Written informed consent was obtained before study enrollment. All experimental procedures were conducted in compliance with the Declaration of Helsinki after full Institutional Review Board approval by the local ethics committee. Eligibility for the study was established based on a TMS safety screening questionnaire and predefined inclusion/exclusion criteria. At the time of the study, the participant patient was taking 10–15 mg once daily of Ditropan for bladder control.

### Surface Electromyography (EMG)

Surface EMG was recorded by single bipolar differential electrodes (MA300-28, Motion Lab Systems Inc., Baton Rouge, LA, USA) from the extensor carpi radialis (ECR) and flexor carpi radialis (FCR) bilaterally. EMG signals were amplified, filtered (10–1,000 Hz), sampled at 2,000 Hz *via* a 1401 plus (Cambridge Electronics Design Ltd., England), and stored for offline analysis.

### Repetitive Transspinal Stimulation for NeuroRecovery

The cervical 5 spinous process was identified *via* palpation, and a single cathode electrode (10.2 cm × 5.1 cm, Uni-Patch™ EP84169, Wabasha, MA, USA) was placed along the vertebrae equally between the left and right paravertebral sides. Due to its size, the electrode covered cervical 5 to thoracic 2 vertebral levels. Two reusable self-adherent electrodes (anode, same type as the cathode), connected to function as a single electrode, were placed bilaterally on the clavicles (10). The cathode and anode electrodes were connected to a constant current stimulator (DS7A, Digitimer, UK) that was triggered by Spike 2 scripts (CED Ltd., UK).

**Figure 1:**
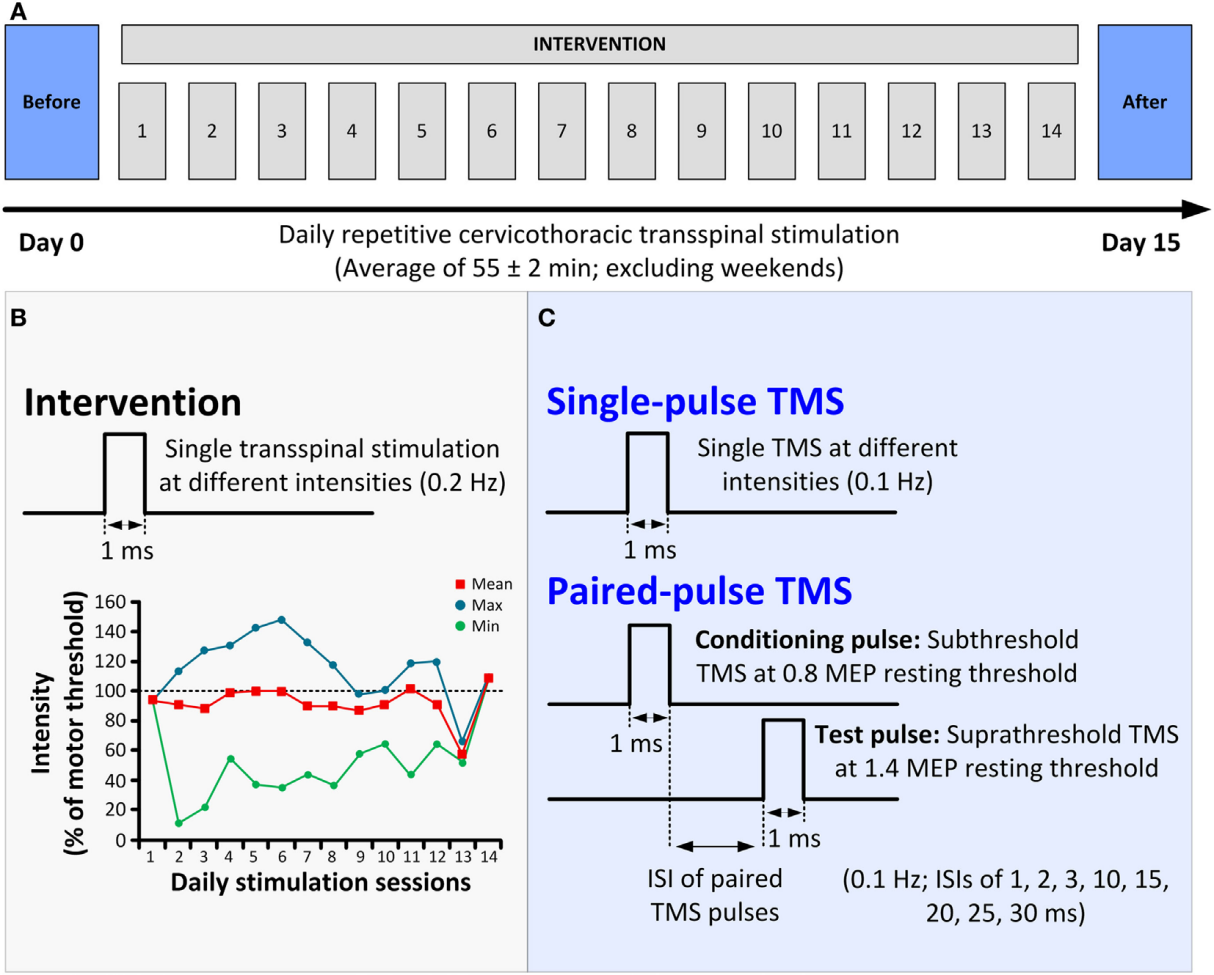
**Protocol of transspinal stimulation for neurorecovery**. **(A)** Repetitive cervicothoracic transspinal stimulation protocol. **(B)** Illustration of single-pulse transspinal stimulation delivered during the intervention, along with the intensities of daily transspinal stimulation normalized to the baseline transspinal-evoked potential motor threshold. **(C)** Illustration of single and paired transcranial magnetic stimulation (TMS) pulses for recording motor-evoked potentials (MEPs). Single-pulse TMS at different stimulation intensities was delivered for assembling the MEP recruitment input–output curves. Paired-pulse TMS was used to condition MEPs at different interstimulus intervals (ISIs) of 1, 2, 3, 10, 15, 20, 25, and 30 ms.

The participant received 14 sessions of repetitive cervicothoracic transspinal stimulation at 0.2 Hz daily for an average of 55 ± 2 min (mean ± SE; 771 min in total; excluding weekends; Figure 1A) while in supine, with hips–knees flexed at 30°. Constant position of the cathodal electrode across sessions was possible by marking the area *via* a Tegaderm microfilm and daily checking of the electrode site based on anatomical landmarks. The stimulation intensities over the course of the intervention ranged from 5 to 68 mA, with an average intensity of 42.5 mA. To avoid exhaustion of spinal motor neurons and facilitate spontaneous depolarization of neurons (17), daily stimulation was delivered in blocks of 10-min during which stimulation intensity ranged from below motor threshold to stimulation intensities that evoked bilateral muscle contractions (Figure 1B).

### Cortical and Corticospinal Excitability Measures

The neurophysiological tests described below were conducted before and 1 day after cessation of repetitive cervicothoracic transspinal stimulation (Figures 1A,C). TMS was delivered *via* a Magstim 200^2^ stimulator (Magstim, UK) with a double-cone coil (diameter 110 mm) according to procedures previously utilized (13–15).

Changes in cortical excitability were established from the right ECR and FCR MEPs recorded in response to paired TMS pulses over the left M1 (Magstim BiStim^2^ module Magstim, UK) with the subject seated. Conditioned MEPs were recorded randomly at the interstimulus intervals (ISIs) of 1, 2, 3, 10, 15, 20, 25, and 30 ms. The conditioning TMS (first stimulus) and the test TMS (second stimulus) were set at 0.8 [=38% maximum stimulator output (MSO)] and 1.4 (=68% MSO) of the targeted ECR MEP resting threshold, respectively. At short ISIs, depression of MEPs has been attributed to intracortical inhibition (ICI) (18, 19), while MEP facilitation at medium-latency ISIs has been attributed to a different population of cortical neurons that are prominent in late indirect (I) waves (20, 21).

The subthreshold conditioning TMS intensity was selected based on absent MEPs in ECR/FCR muscles bilaterally, while the suprathreshold test TMS intensity was selected based on the known strength of ICF as a function of the MEP size (22) and ensuring that the test ECR/FCR MEPs were ~50% of the corresponding maximal MEP. Test and conditioned MEPs were recorded before and after repetitive transspinal stimulation at exactly the same stimulation intensities. Under control conditions, 24 test MEPs were recorded at 0.1 Hz. Under subthreshold conditioning TMS, 12 MEPs were recorded at 0.1 Hz for each ISI.

Changes in corticospinal excitability were assessed from the right ECR/FCR and left ECR MEP recruitment input–output curves, which were assembled with single TMS pulses in ascending order from stimulation intensities that MEPs were absent until maximum amplitudes were obtained. At least five MEPs at 0.1 Hz were recorded at each stimulus intensity. MEP recruitment curves were assembled with the same intensities before and after repetitive cervicothoracic transspinal stimulation.

Voluntary muscle strength, sensation, and spasticity were also evaluated *via* standardized clinical tests.

### Data Analysis

Motor-evoked potentials were measured as the area of the full-wave-rectified EMG signals (Spike 2, CED Ltd., UK). MEP latencies were measured based on the cumulative sum calculations (23) by defining the precise turning point post-stimulus while taken into consideration the pre-stimulus EMG for 60 ms. The cumulative sum calculations were applied to the waveform average of resting test MEPs.

The MEPs evoked upon paired TMS pulses at different ISIs were normalized to the homonymous mean size of the test MEP. The Shapiro–Wilk test was performed to test data for normal distribution. A Kruskal–Wallis one-way analysis of variance (ANOVA) was performed to determine differences of conditioned MEPs from the test MEPs. A two-way ANOVA was performed to determine the effect of time (before vs. after) and ISI of paired TMS pulses on the conditioned MEPs. Holm–Sidak *t*-tests for multiple comparisons were used to test for significant interactions between these two factors.

The mean-rectified size of the right ECR/FCR and left ECR MEPs was estimated and plotted against each MSO. This was done separately for MEPs recorded before and after repetitive transspinal stimulation. A two-way ANOVA was applied separately to MEPs recorded from left or right wrist muscles to establish statistically significant differences between time and stimulation intensity. A Boltzmann sigmoid function (SigmaPlot 11, Systat Software Inc.) was also fitted to the MEP recruitment input–output curves separately (10). In all tests, statistical significance was assumed when *p* < 0.05. Data are presented as mean ± SE in the text and figures.

## Results

**Figure 2:**
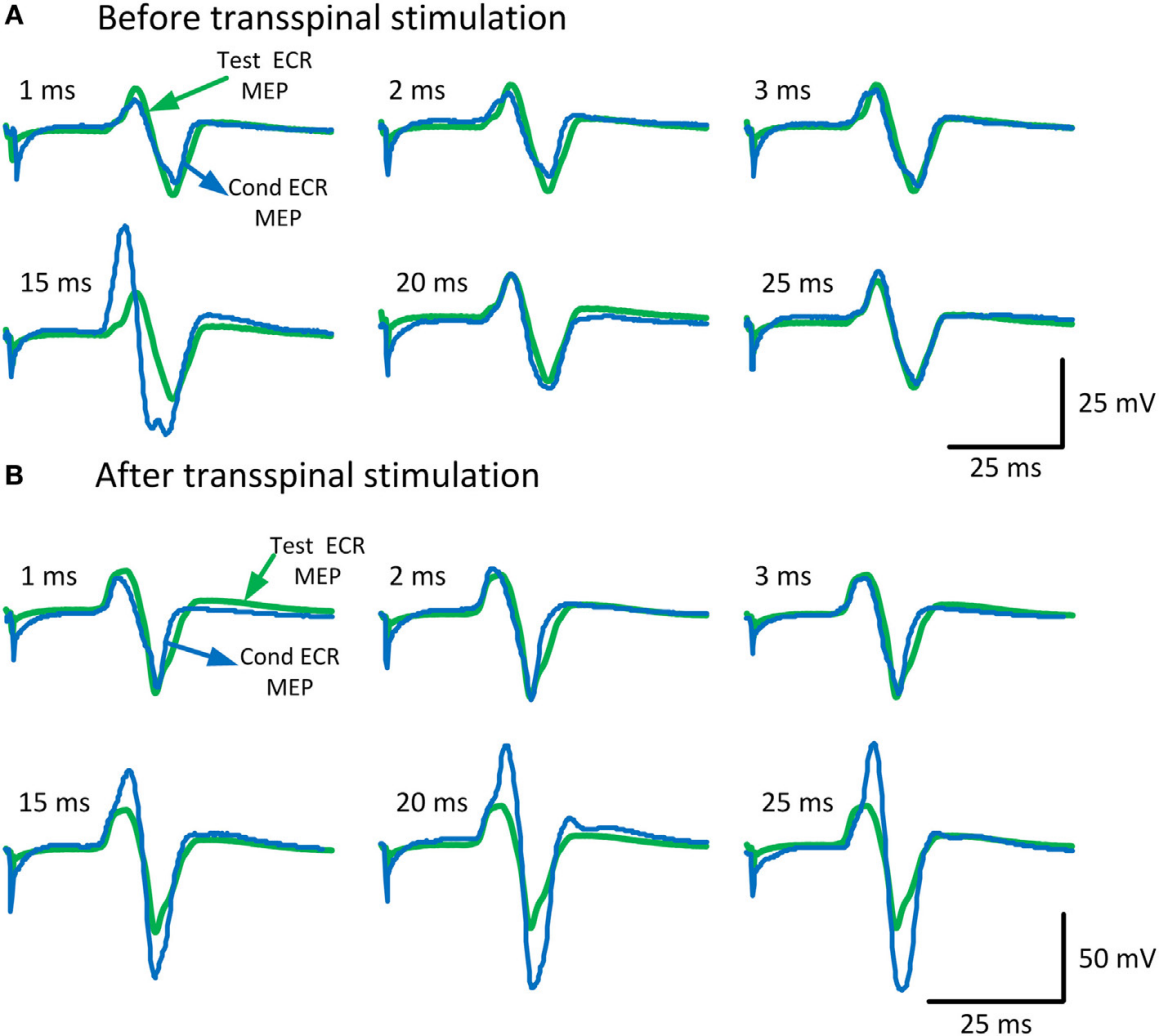
**Conditioned motor evoked potentials (MEPs) of the right arm**. MEPs tested in the resting extensor carpi radialis (ECR) muscle upon single- and paired-pulse transcranial magnetic stimulation (TMS) before **(A)** and after **(B)** repetitive cervicothoracic transspinal stimulation. Traces show the averages of 24 test MEPs (green traces) and 12 conditioned MEPs (blue traces) for short- and medium-latency interstimulus intervals.

To characterize changes in cortical excitability, the amount of ICI and ICF before and after repetitive cervicothoracic transspinal stimulation was assessed *via* paired TMS pulses delivered to the left M1. Figure 2 illustrates the average of test (green traces) and conditioned (blue traces) MEPs in the right resting ECR muscle. Note the strong MEP facilitation at medium ISIs after repetitive transspinal stimulation (Figure 2B).

**Figure 3:**
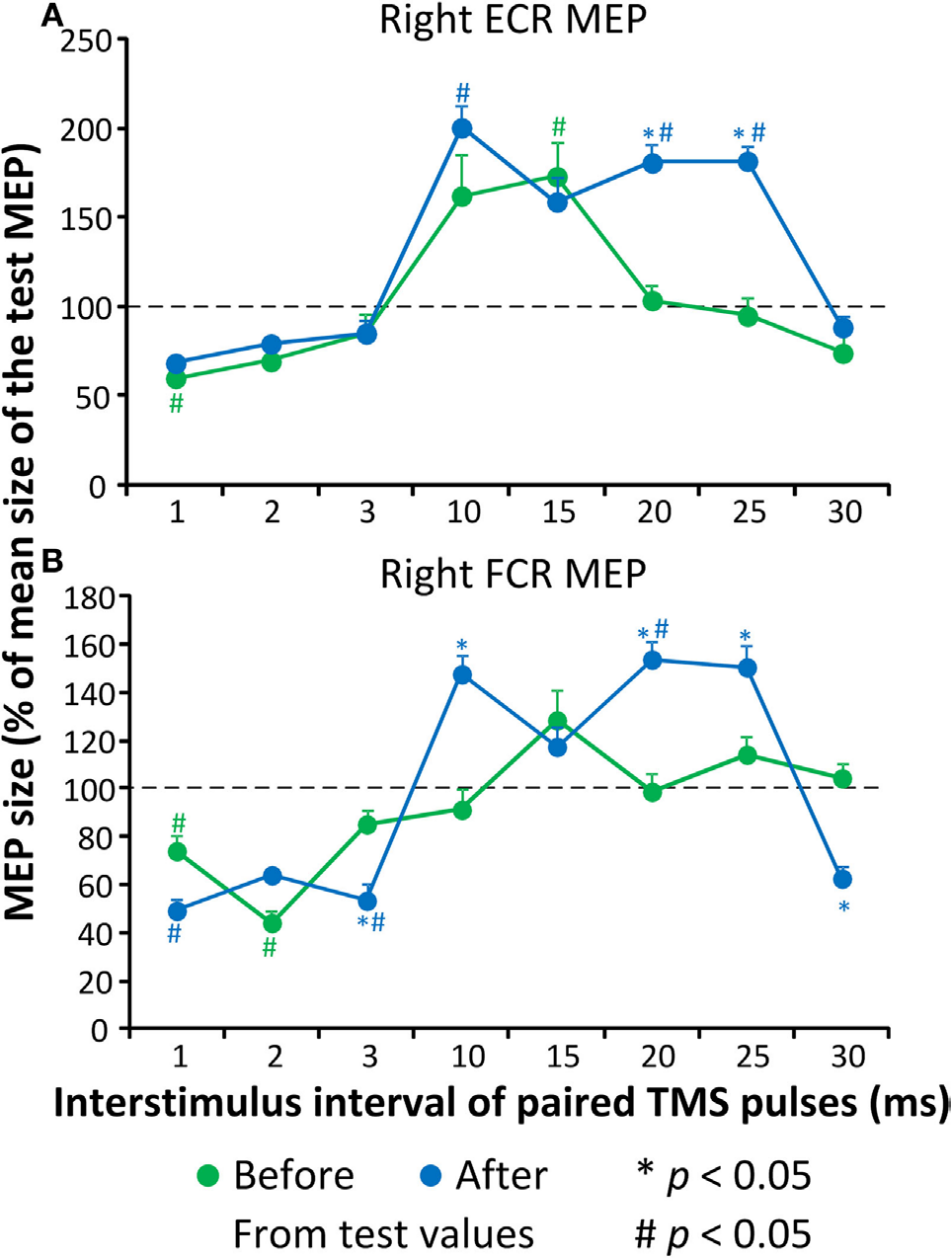
**Cortical excitability measures before and after repetitive cervicothoracic transspinal stimulation**. Overall amplitude of extensor carpi radialis (ECR) **(A)** and flexor carpi radialis (FCR) **(B)**. Motor-evoked potentials (MEPs) from the right arm upon paired-pulse transcranial magnetic stimulation (TMS). Conditioned MEPs are presented as a percentage of the mean size of the homonymous test MEP. Error bars indicate SE. *p < 0.05 for before–after comparisons, ^#^*p* < 0.05 from homonymous test MEP values.

Before repetitive cervicothoracic transspinal stimulation, the conditioned right ECR MEP was significantly different from the test MEP values at the ISIs of 1 and 15 ms (Kruskal–Wallis one-way ANOVA on ranks, Figure 3A), suggesting presence of ICI and ICF at these intervals. After repetitive transspinal stimulation, a significant effect of time [*F*_(1)_ = 15.75, *p* < 0.001] and ISI of paired TMS pulses [*F*_(7)_ = 32.66, *p* < 0.001], and a significant interaction between time and ISI [*F*_(7)_ = 8.53, *p* < 0.001] were found on the conditioned right ECR MEP amplitudes. Based on Holm–Sidak multiple comparisons, the conditioned right ECR MEPs were significantly different before and after repetitive transspinal stimulation (Figure 3A) at the ISIs of 20 and 25 ms, suggesting potentiation of ICF. The conditioned right FCR MEP before repetitive transspinal stimulation was different from the test MEP values at the ISIs of 1 and 2 ms (Kruskal–Wallis one-way ANOVA on ranks, Figure 3A), suggesting present ICI and absent ICF. After repetitive transspinal stimulation, a significant effect of time [*F*_(1)_ = 4.43, *p* = 0.037] and ISIs [*F*_(7)_ = 43, *p* < 0.001] and a significant interaction between these two factors [*F*_(7)_ = 13.75, *p* < 0.001] were found on the conditioned right FCR MEPs. The conditioned FCR MEPs were significantly different before and after repetitive transspinal stimulation at the ISIs of 3, 10, 20, 25, and 30 ms (Figure 3B).

**Figure 4:**
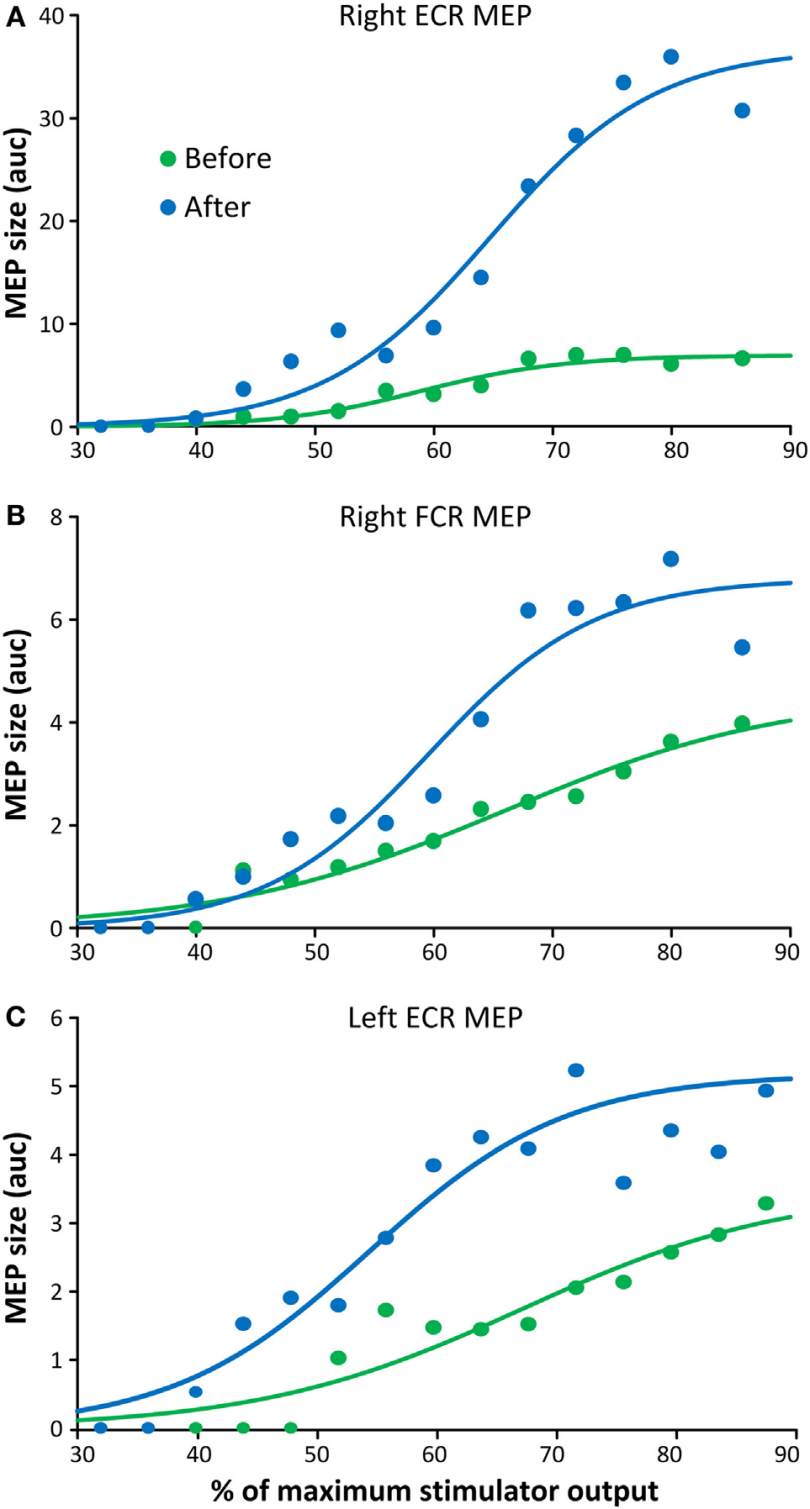
**Corticospinal excitability measures before and after repetitive cervicothoracic transspinal stimulation**. Motor-evoked potentials (MEPs) recorded from the right extensor carpi radialis (ECR) **(A)**, right flexor carpi radialis (FCR) **(B)**, and left ECR **(C)** muscles before and after repetitive transspinal stimulation are depicted as the area under the curve (auc) and are plotted against the percentage of the maximum stimulator output. Before and after repetitive transspinal stimulation, MEP recruitment input–output curves were assembled with single-pulse transcranial magnetic stimulation (TMS) at exactly the same stimulation intensities. A sigmoid fit to the data is also shown. Note the significant increases in MEP sizes after repetitive transspinal stimulation regardless of the stimulation intensity.

The latency of the right ECR, right FCR, and left FCR MEP before repetitive cervicothoracic transspinal stimulation was 20.33, 20.47, and 20.93 ms, while after stimulation the latencies were 18.6, 18.11, and 19.17 ms, respectively. The MEPs, recorded from the right ECR (Figure 4A), right FCR (Figure 4B), and left ECR (Figure 4C) muscles at different stimulation intensities plotted against the percentage of the MSO (recruitment input–output curves), clearly demonstrate that repetitive transspinal stimulation increased corticospinal excitability. MEPs were absent in the left FCR muscle. The right ECR MEPs were significantly different before and after repetitive transspinal stimulation [*F*_(1,13)_ = 15.34, *p* = 0.002], a result found also for the recruitment curves of the right FCR [*F*_(1,15)_ = 15.2, *p* = 0.002] and left ECR [*F*_(1,15)_ = 36.04, *p* < 0.001] MEPs.

The clinical outcomes before and after repetitive cervicothoracic transspinal stimulation regarding voluntary motor strength and sensation are reported in Table 1. The participant reported that during the intervention he started to sweat in the upper back and armpits, a response that had stopped after the injury. After repetitive cervicothoracic transspinal stimulation, self-reported frequency and severity of spasms in the legs decreased by 33% (Penn Spasm Frequency Scale), and ankle clonus decreased from slight to no resistance at increased velocities of passive movement (Tardieu Scale). Lastly, the participant reported that his legs felt less tense and more relaxed.

**Table 1:** **Clinical outcomes**.

AIS UEMS (0–25)				AIS LT (0–56)				AIS PP (0–56)			
Right		Left		Right		Left		Right		Left	
Before	After	Before	After	Before	After	Before	After	Before	After	Before	After
12	17	12	15	32	32	31	31	16	22	16	22

*AIS, American Spinal Injury Association Impairment Scale; UEMS, upper extremity motor score; LT, light touch; PP, pin prick*.

## Discussion

Repetitive cervicothoracic transspinal stimulation remodeled cortical activity acting on distal extensor and flexor wrist muscles in one person with chronic cervical motor incomplete SCI. Further, corticospinal excitability increased regardless of the levels of stimulation intensities and arm side, suggesting for bilateral strengthening of corticospinal connections. These results support our hypothesis and are consistent with the cortical activity reported after spinal cord stimulation (24).

In the cervical SCI participant, we found that after repetitive cervicothoracic transspinal stimulation ICF increased in both extensor and flexor wrist muscles and ICI increased at 3 ms in the wrist flexor muscle (Figure 3). In people with cervical SCI, the silent period of MEPs and ICI in small hand muscles is significantly reduced (25). A critical question is which neuronal pathways were involved in the remodeling of cortical excitability after repetitive transspinal stimulation. Impaired activity of ascending proprioceptive sensory pathways has been linked to reduced activity of cortical inhibitory interneuronal circuits (26). In healthy control subjects, we recently demonstrated that one session of repetitive transspinal stimulation alters the afferent-mediated MEP facilitation (15). Consequently, repetitive transspinal stimulation in the participant with cervical SCI could have potentially altered the cortical integration of ascending sensory inputs from the spinal cord.

After repetitive cervicothoracic transspinal stimulation, the latencies of MEPs recorded from wrist flexor/extensor muscles decreased by 1.7 and 2.3 ms, while the amplitude of MEPs recorded from both upper limbs increased regardless the stimulation intensity (Figure 4). Decrements in MEP latencies suggest faster conduction velocities of corticospinal axons as they pass through the site of the spinal cord lesion (27). The increased MEP amplitudes at varying stimulation intensities in both upper limbs can be attributed to remodeling of cortical maps and increased corticospinal drive after repetitive transspinal stimulation. MEPs can capture organization of motor cortical maps, which are pathological as early as 6 days after cervical SCI (28, 29). MEPs evoked at high intensities are likely to evoke both direct and multiple indirect waves making the summation of signals in the spinal cord more easily compared to lower TMS intensities. However, the MEPs, especially those recorded from the left ECR muscle, increased at very low stimulation intensities (Figure 4C). One possible explanation is that repetitive transspinal stimulation enabled spinal motoneurons to reach depolarization threshold at lower stimulation intensities, making them more excitable. However, the possibility of changes in indirect waves cannot be disregarded. A potential mechanism that could account for the pronounced increase in MEP sizes after repetitive transspinal stimulation is long-term potentiation-like mechanisms (30).

The neurophysiological changes coincided with improvements in volitional muscle strength, sensation, self-reported reduced frequency and severity of spasms in the legs, and reversal of anhidrosis below the lesion level. Thermoregulation *via* sweating is intimately linked to direct autonomic control *via* hypothalamic regulation. Peripheral cold and warm receptors project to the hypothalamus *via* the sympathetic ganglia at the spinal cord (31), while hypothalamic connection to the spinal sympathetic circuits is greatly impaired in cases of lesions above thoracic 6 (32). The increased blood flow to the skin and muscle after transspinal stimulation is abolished after dissection of ventral roots, bilateral lumbar sympathectomy performed 1 week before stimulation, and is prevented by pharmacological blockade of autonomic transmission at the neuroeffector junction (33–35). Thus, repetitive transspinal stimulation could have potentially affected thermoregulation by changing the blood flow in skin and muscle as well as by excitation of the sympathetic trunk and associated ganglia directed to the sweat glands.

### Limitations

There are several limitations to this study. First, we did not establish whether the observed neural changes were transferrable and meaningful by improving the ability of hand/wrist function in daily motor activities. A second limitation is that spasticity was assessed *via* standard clinical tests and not by objective methods involving surface EMG activity of antagonist muscles in response to imposed passive movement at different velocities. Finally, transspinal stimulation is a non-specific neuromodulation paradigm that makes it difficult to define the primary spinal pathways and circuits mediating neuronal changes. However, it is non-invasive, cost-effective, and safe for people with and without SCI. It would be important in future studies to assess spasticity *via* objective methods, establish to what extent the observed neurophysiological changes are transferrable in daily motor activities, and perform complex simulation studies to delineate the neuronal mechanisms and pathways underlying plasticity after repetitive transspinal stimulation.

## Conclusion

Repetitive cervicothoracic transspinal stimulation remodeled cortical and corticospinal activity, reversed anhidrosis, reduced the frequency and severity of spasms, and ankle clonus in a person with cervical motor incomplete SCI. The neural changes may improve the ability to perform daily activities and thus improve quality of life in these patients. Repetitive transspinal stimulation can be utilized as a therapeutic intervention to promote neuroplasticity and recovery of motor function. Our findings thus open new avenues for targeted brain plasticity in neurological disorders.

## Ethics Statement

This study was carried out in accordance with the recommendations of the Biomedical Institutional Review Board of the City University of New York (New York, NY, USA). The subject gave written informed consent in accordance with the Declaration of Helsinki. The protocol was approved by the City University of New York (New York, NY, USA).

## Author Contributions

MK designed the study. LM and MK performed the neurophysiologic assessments. LM delivered the stimulation. MK processed the neurophysiologic data, interpreted the data along with LM, performed the statistical analyses, and wrote the first draft of the manuscript. Both MK and LM approved the final submitted manuscript.

## Conflict of Interest Statement

The authors declare that the research was conducted in the absence of any commercial or financial relationships that could be construed as a potential conflict of interest.
